# Gut Microbiota and Nutrition in Nursing Homes: Challenges and Translational Approaches for Healthy Aging

**DOI:** 10.1016/j.advnut.2025.100520

**Published:** 2025-09-17

**Authors:** Cristina Jiménez-Arroyo, Natalia Molinero, M Victoria Moreno-Arribas

**Affiliations:** Institute of Food Science Research (CIAL), CSIC-UAM, Madrid, Spain

**Keywords:** institutionalized elderly, gut microbiome, diet–microbiota interactions, healthy aging, long-term care, microbiome-informed strategies, nutritional interventions

## Abstract

The growing aging population is leading to an increase in demand for long-term care. This is particularly true in nursing homes, where residents are exposed to various challenges such as immunosenescence, frailty, multimorbidity, and dietary and environmental constraints. These interrelated factors contribute to gut microbiota alterations, underscoring the need for tailored strategies to preserve health and resilience in a long-term care setting. Despite its recognized relevance in healthy aging, the gut microbiome of institutionalized elderly remains markedly understudied. This review provides a comprehensive report of the current evidence on the interplay between diet, gut microbiota, and aging among nursing-home residents. The available literature suggests that both aging and institutional living contribute to a less favorable microbiome profile, and several contributing factors, many of them dietary, have been identified. Altered gastrointestinal physiology, malnutrition, and other common conditions in residential care, such as functional or cognitive impairments, frequently lead to changes in food intake that affect the gut ecosystem. Decline in immune system, increased infection risk, sarcopenia, cognitive deterioration, and high medication burden have also been linked to microbiota disruptions in this population. Importantly, adjusting several modifiable features of institutional care, particularly those related to diet and lifestyle, may help counteract these effects by supporting gut health. We further examine how appropriate nutritional strategies can positively influence gut microbiota composition and function, offering a pathway to promote resilience and functionality even in the presence of geriatric syndromes. In addition to identifying these challenges, this review outlines feasible, microbiota-informed strategies to improve quality of life and health outcomes. These include individualized dietary adaptations, targeted supplementation, physical activity interventions, and the integration of digital and artificial intelligence tools to support personalized nutrition. Finally, we highlight the need for standardized protocols and implementation science frameworks to enhance clinical translation, thereby advancing an integrative and as yet underrepresented perspective on microbiota-based strategies to promote healthier aging trajectories in institutionalized elderly.

**Statement of Significance**

This review is the first to comprehensively integrate diet–microbiota interactions in the context of institutionalized elderly to provide geriatric, nutritional, and microbial evidence while proposing feasible multimodal strategies to enhance resilience and support healthy aging in a nursing-home setting.

## Introduction

The population is aging at an unprecedented pace, particularly in developed countries. According to the WHO, the number of people aged ≥60 y is expected to rise from 1 billion in 2019 to 1.4 billion by 2030 and to 2.1 billion by 2050. This estimated demographic shift underscores the urgent need for strategies and public policies to promote active and healthy aging while minimizing the risks of frailty and dependency [1,2].

From a biological perspective, aging refers to the progressive accumulation of molecular and cellular damage that then critically affects key homeostatic systems, such as the nervous, immune, and endocrine systems, and their bidirectional communication [3]. This gradual deterioration leads to physical and cognitive decline, increasing risk of disease and mortality [4]. Importantly, the negative effects of aging on health are not experienced uniformly across individuals. Although some people present with delayed decline and healthy aging, others show accelerated deterioration, becoming more susceptible to conditions such as frailty and cognitive impairment.

Although numerous theories have been proposed to explain the aging process, López-Otín et al. [5] identified 9 primary hallmarks, including genomic instability, telomere attrition, epigenetic alterations, loss of proteostasis, dysregulated nutrient sensing, mitochondrial dysfunction, cellular senescence, stem cell exhaustion, and altered intercellular communication. More recently, 3 additional hallmarks have been recognized—impaired macroautophagy, chronic inflammation, and gut dysbiosis. These highlight microbiome as a critical factor in healthy aging [6,7].

An aging population is closely associated with an increased demand for long-term care, particularly in nursing homes. These institutions provide specialized services to address the complex needs of elderly; however, they may also affect residents’ physical, mental, and social well-being. On the one hand, nursing homes offer a safe environment with continuous care [8]; on the other hand, the transition to institutionalized living can be accompanied by a decline in quality of life, loss of autonomy, and negative psychological outcomes such as diminished personal identity [9,10]. These characteristics make nursing homes a unique setting that can either alleviate or intensify the challenges of aging.

There are increasing concerns about the diet and nutrition of elderly, who are particularly vulnerable to undernutrition—a condition that significantly impacts both quality of life and life expectancy, especially in institutionalized populations. Recently, gut microbiota has emerged as a key player in the regulation of host health [11] and is now a major focus in aging research. However, few review articles till date have specifically focused on the institutionalized elderly, despite the fact that this population often presents overlapping characteristics, including frailty, increased susceptibility to infections, multimorbidity, and the concurrent use of multiple medications.

Although the gut–brain axis has gained prominence as a tractable target for supporting brain health in elderly through dietary and microbial modulation [12], the literature on diet–microbiota interactions and interventions in a nursing-home setting has yet to be systematically reviewed. Moreover, a holistic perspective that fully integrates the unique challenges and potential opportunities within this research framework remains lacking.

To address these gaps, this review explores the complex interactions between diet, gut microbiota, and aging among institutionalized elderly—an often overlooked but highly vulnerable population. We examine how physiological, clinical, nutritional, and environmental factors inherent in long-term care settings shape the gut microbiota composition and function. We also identify the challenges to and opportunities for intervention and propose feasible, microbiota-informed, multimodal strategies to promote health, resilience, and functional autonomy among nursing-home residents.

## Gut Microbiome Changes Associated with Aging: Impact of Place of Residence, Nursing Homes, and Long-Term Care

### The aging gut microbiome

The gut microbiome undergoes significant changes with age by adapting and responding to environmental signals, much like host cellular systems. Beyond genetic determinants, the composition and function of the gut microbiota are physiologically influenced by a range of lifestyle factors from birth to old age, including social and environmental conditions, dietary habits, and physical activity [13,14]. Although multiple aging trajectories in the human gut microbiome have been described, aging is frequently associated with decreased bacterial diversity and increased interindividual variability, which may reflect an imbalance in gut homeostasis that is referred to as dysbiosis. Dysbiosis often involves a reduction in short-chain fatty acid (SCFA)-producing species such as *Faecalibacterium prausnitzii*, *Eubacterium* spp., and *Bifidobacterium* spp., along with an increased abundance of potentially harmful bacteria, including *Clostridioides difficile* and members of the *Enterobacteriaceae*, *Streptococcaceae*, and *Staphylococcaceae* families—groups linked to inflammation and reduced microbial stability [15]. Interestingly, recent studies suggest that the shift from dominant core taxa (e.g., *Bacteroides* spp.) toward rarer or minority microorganisms represents a compensatory adaptation to the physiological changes associated with aging [16]. Medication history and clinical diagnoses also account for a significant portion of variation in the gut microbiome among elderly, underscoring the impact of health status, lifestyle, and lifetime exposures [17,18].

Growing interest in this field has prompted new research approaches that aim to determine whether age-associated microbiota changes can indicate healthy or unhealthy aging trajectories [15,19]. Longitudinal studies have linked lower microbial diversity to increased comorbidities and diminished immunocompetence [15]. In contrast, healthy centenarians tend to harbor unique microbiomes with higher diversity and a greater abundance of beneficial taxa such as *Christensenellaceae*, *Akkermansia*, *Lactobacillus*, and *Bifidobacterium* spp. [20]. However, some SCFA producers, such as *Coprococcus* sp. and *F. prausnitzii*, appear to decline in centenarians, whereas certain potentially pathogenic genera, including *Eggerthella* and *Enterobacteriaceae*, are more prevalent in the oldest individuals [[21], [22], [23]]. Several studies have also reported significant associations between specific bacterial taxa and clinical or lifestyle variables in centenarians, particularly among those adhering to a Mediterranean diet [24,25].

In addition to compositional shifts, age-associated changes in gut microbial metabolic activity have also been described. These include increased proteolytic capacity, resulting in greater production of potentially toxic metabolites from protein fermentation. Conversely, the generation of beneficial compounds such as indolepropionate, a product of tryptophan metabolism, has been positively associated with longevity in centenarians [26,27]. Moreover, microbial metabolism of specific secondary bile acids, such as isoforms of lithocholic acid, has been implicated in reducing susceptibility to pathobiont infections, potentially contributing to the preservation of intestinal homeostasis in the elderly [28].

### Impact of place of residence on the aging gut microbiome

Place of residence appears to be a key determinant of gut microbiome composition and functionality in elderly [29]. Although this remains a relatively underexplored area, several studies have identified notable differences in the microbial composition of individuals residing in nursing homes and those living in the community. For instance, Claesson et al. [30] reported that nursing-home residents had significantly less diverse gut microbiota, with higher proportions of microorganisms belonging to the phylum *Bacteroidota* and genera such as *Parabacteroides*, *Eubacterium*, *Anaerotruncus*, *Lactonifactor*, and *Coprobacillus.* In contrast, community-dwelling elders exhibited higher levels of the phylum Bacillota and the family *Lachnospiraceae*. These differences were associated with greater frailty, lower muscle mass, and poorer overall health status among institutionalized individuals [30].

The length of stay in a long-term care facility also appears to influence the gut microbiome. During the first year of institutionalization, elderly residents often experience microbial dysbiosis. However, after this initial phase, the microbiome may reach a relatively stable and resilient state [31,32]. Residents institutionalized for >1 y were found to harbor elevated levels of putatively inflammation-associated species, such as *Bacteroides vulgatus* and *Veillonella parvula*, along with beneficial or symbiotic species, such as *F. prausnitzii*, *Eubacterium* spp., *Ruminococcus bromii,* and *R. lactaris* [31]. The same study reported an early increase in pathogenic species—including *Enterococcus faecalis*, *Haemophilus parainfluenzae*, and *Klebsiella pneumoniae*—as well as a decline in anti-inflammatory taxa during the first 6 mo.

Similarly, Jeffery et al. [32] found that microbiome changes related to frailty occurred gradually over the first 18 mo of institutionalization. Importantly, individuals with lower microbial diversity initially were more susceptible to pronounced shifts in their microbiota over timeHaga clic o pulse aquí para escribir texto..

Despite this emerging evidence, our understanding of how residential setting influences the gut microbiome in elderly remains limited. The complex interplay of factors inherent to long-term care environments, including nutrition, clinical care, physical activity, and medication use, warrants further investigation so as to design strategies to optimize microbiome health in this vulnerable population.

## Diet–Microbiota Interactions in Institutionalized Elderly

### Physiological changes associated with aging and their impact on nutrition

There is strong scientific evidence supporting the role of adequate nutritional status in maintaining health and preventing the onset and progression of chronic diseases, particularly among elderly. Nutritional requirements change throughout life owing to physiological alterations, including changes in body composition, basal metabolic rate, and nutrient absorption. These changes are often accompanied by other age-related lifestyle shifts such as sedentary behavior, loneliness, or depression [33].

**FIGURE 1:**
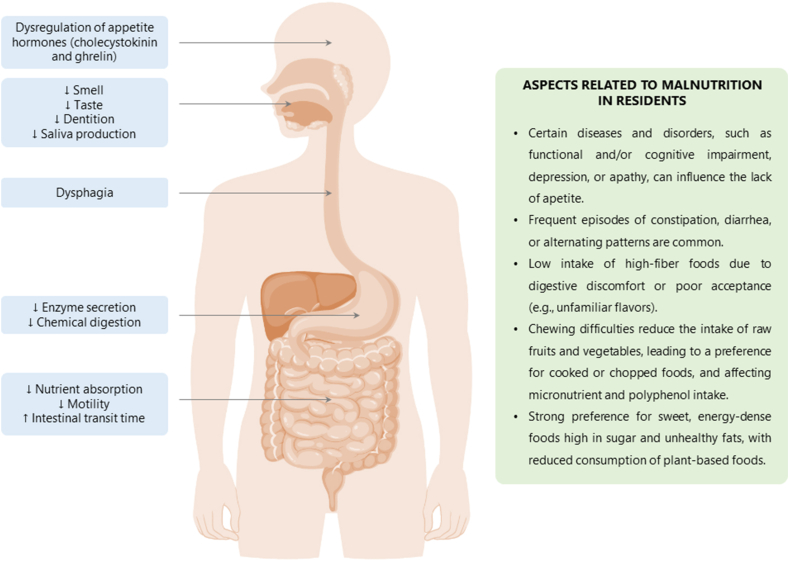
Age-related physiological changes in the gastrointestinal tract and other factors influencing nutritional status and food choices in institutionalized elderly.

Aging induces physiological changes that affect gastrointestinal function and, consequently, dietary patterns (Figure 1). In the oral cavity, a decline in taste and smell sensitivity, reduced chewing efficiency because of tooth loss, and decreased saliva production can impair swallowing [15,34]. In some cases, these changes contribute to dysphagia, defined as the difficulty or inability to form and move food bolus safely from the mouth to the esophagus [35].

At the gastrointestinal level, reduced secretion of digestive enzymes (e.g., pepsin, lipase, chymotrypsin, and amylase) affects chemical digestion [36]. In the intestine, motility and nutrient absorption decline, and transit time increases, potentially affecting both the composition and activity of the gut microbiota [34,36]. Although several animal studies suggest that aging impairs the gut barrier function, a recent study in humans did not confirm this hypothesis [37].

Hormonal dysregulation, including changes in cholecystokinin and ghrelin levels, also contributes to reduced appetite, influencing both food intake and food preferences [36]. In addition, sensory impairments (e.g., taste and smell loss) may cause certain foods to become unappealing, further altering eating behavior [38]. Together, these changes can affect not only food choices and dietary habits but also the quantity and quality of nutrients ingested, leading to deficiencies in essential vitamins and micronutrients and increasing risk of malnutrition [34,39]. These nutritional imbalances may, in turn, further influence the gut microbiota, potentially exacerbating microbial dysbiosis.

Institutionalized elderly are at particularly high risk of malnutrition owing to the combined influence of age-related physiologic decline and environmental factors. Additional contributing factors include functional and cognitive impairment, apathy, and especially depression, which is considered one of the most significant and modifiable risk factors [41]. However, the prevalence of malnutrition in geriatric care facilities is difficult to determine, with estimates ranging from 1.5% to 66.5% [40].

In nursing homes, malnutrition frequently presents as undernutrition. It is estimated that >50% of new residents have a BMI of <23 kg/m^2^, and 15% experience recent weight loss [42]. Indeed, institutionalized elderly tend to have lower BMIs, likely because of chronic nutritional deficiencies, making them more vulnerable to malnutrition, cognitive decline, and impaired physical performance than their community-dwelling counterparts [43]. However, overweight and obesity are also prevalent in these settings and have been associated with a higher risk of institutionalization [44,45]. In the United States, the prevalence of obesity (BMI >30 kg/m^2^) among nursing-home residents increased from 22% in 2005 to 28% in 2015 [46].

Moreover, the quality of diet provided in long-term care facilities substantially varies with the geographic location, with marked differences in nutritional care practices across European countries [47]. Although each country—and often each region—establishes dietary recommendations and nutritional requirements tailored to the needs of elderly, evidence suggests that these guidelines are not always properly adhered to, leaving nutritional needs unmet [42,48,49].

### Nutritional features and the gut microbiota in institutionalized populations

Diet is one of the most influential factors that can modulate the diversity and functionality of the gut microbiome [15,19]. Among dietary patterns, the Mediterranean and Western diets have been the most extensively studied for their impact on microbiota composition and associated health outcomes. The Western dietary pattern—characterized by high consumption of red and processed meats, prepackaged foods, sweets, fried foods, refined grains, saturated fats, and sugar-sweetened beverages—has been associated with metabolic disorders, chronic inflammation, and a less diverse gut microbiota [50].

In elderly, detailed dietary assessments revealed clear differences based on the place of residence. Individuals living independently tend to consume diets rich in fruits, vegetables, and fiber and lower in fat. In contrast, elderly residents in nursing homes often consume diets that are low in fiber; such diets are associated with a higher abundance of potentially harmful bacterial groups (e.g., *Proteobacteria* and *Clostridia*) and a reduced presence of beneficial taxa such as *Lactobacillus* and *Bifidobacterium* [30].

Preclinical and clinical studies have consistently shown that the Mediterranean diet—rich in whole grains, legumes, fruits, vegetables, nuts, and olive oil—is associated with healthier aging and promotes a diverse, beneficial microbiome [15,51]. The NU-AGE study ('New dietary strategies addressing the specific needs of the elderly population for healthy ageing in Europe'), a 1-y dietary intervention conducted in 5 European countries, demonstrated that adherence to a personalized Mediterranean diet led to significant improvements in the gut microbiota of elderly. Notably, it increased the abundance of health-associated bacteria such as *F. prausnitzii*, *Roseburia*, and *Eubacterium* [15]. More recent findings indicate that a 1-y lifestyle intervention combining the Mediterranean diet with regular physical activity beneficially modulates the gut microbiota in elderly with obesity and metabolic syndrome [52].

In addition to its metabolic benefits, the Mediterranean diet has been associated with neuroprotective effects [12,53]. Key dietary components, including PUFAs, polyphenols, and antioxidant vitamins such as vitamins C, E, B_12_, and folate, have been associated with a reduced risk of neurodegenerative diseases [54]. Furthermore, this dietary pattern may improve the biomarkers of healthy aging in urine and blood [55], increase the production of beneficial microbial metabolites, and reduce intestinal inflammation [25,56].

Other dietary patterns, such as vegan diets, have gained attention not only for their environmental sustainability but also for their influence on human health via the diet–microbiota axis. A recent large-scale study by Fackelmann et al. [57] involving >21,000 individuals from 5 international cohorts enabled an unprecedented metagenomic characterization of vegan, vegetarian, and omnivorous dietary patterns. The findings showed that vegan diets are associated with gut microbiome profiles rich in butyrate-producing and fiber-degrading bacteria, which are correlated with favorable cardiometabolic markers [57]. These results reinforce the potential of well-designed plant-based diets to support a healthy gut microbiome, a particularly relevant consideration in elderly.

However, because of the physiologic changes that occur with aging, vegan diets must be carefully planned in this population to avoid critical nutrient deficiencies, such as vitamin B_12_, as highlighted in previous studies [58]. Therefore, tailoring plant-based interventions for elderly requires balancing nutritional adequacy with strategies that promote a beneficial gut microbiome.

Beyond overall dietary patterns, research into specific dietary components also offers valuable insights into how individual nutrients and bioactives shape the gut microbiota and influence healthy aging, particularly among institutionalized populations.

#### Macronutients

##### Dietary proteins

Proteins are essential dietary components, especially in aging populations, as they are the primary source of essential amino acids required for neurotransmitter synthesis and proper brain function. However, inadequate protein intake is a major concern among institutionalized elderly and a key contributor to protein–energy malnutrition [59]. Low protein intake has been associated with sarcopenia and frailty—conditions that are also linked to alterations in the gut microbiota [60,61]. Undigested proteins that reach the colon can promote the growth of proteolytic bacteria such as *Bacteroides* and *Clostridium* that can produce potentially toxic metabolic byproducts [62]. Conversely, diets rich in plant-based proteins have been associated with greater microbial diversity and increased production of SCFAs [63]. In institutionalized residents, supplementation with protein and fiber led to improvements in nutritional status and intestinal function [64]. In contrast, diets high in animal-based proteins may increase the abundance of *Bacteroides*, which plays a role in protein degradation, while decreasing beneficial taxa such as *Roseburia*, *Blautia*, and *Bifidobacterium longum* [65].

##### Dietary lipids

Lipids are macronutrients essential for cellular structure, energy metabolism, and signaling processes; they also directly affect the gut microbiota. Lipids include SFAs such as palmitic acid, MUFAs such as oleic acid, and PUFAs such as α-linolenic acid [omega-3 (n-3)] and linoleic acid (ω-6) as well as phospholipids and sterols. Although most dietary lipids are absorbed in the small intestine, a small proportion reaches the colon and interacts with the gut microbiota therein.

Diets rich in SFAs have been associated with reduced microbial diversity and an increased abundance of opportunistic pathogens such as *Bilophila wadsworthia* and *Erysipelotrichaceae* [66,67]. Excessive intake of SFAs and trans fats has also been linked to the promotion of a proinflammatory microbiota, characterized by reduced populations of butyrate-producing bacteria and increased abundance of *Pseudomonadota*, further elevating risk of metabolic disorders [68,69]. In contrast, MUFAs—abundantly found in olive oil, a central component of the Mediterranean diet—have anti-inflammatory effects and are associated with beneficial microbial profiles, including an increased abundance of *Akkermansia muciniphila* and other taxa linked to metabolic health. MUFAs may also help reduce intestinal inflammation and improve mitochondrial function [70].

PUFAs, particularly the ω-3 fatty acids, DHA and EPA, as well as ω-6 fatty acids, have been shown to support the growth of beneficial bacteria, including *Bifidobacterium* and *Lactobacillus*, while reducing the abundance of pathogens such as *Escherichia coli* [[71], [72], [73]]. Moreover, PUFAs modulate inflammation through interaction with SCFAs and the regulation of intestinal permeability. Therefore, the quality and composition of dietary fats in the long-term care setting are critical for maintaining gut health and mitigating chronic inflammation. However, evidence suggests that institutional diets often fail to meet the recommendations for healthy fat intake, potentially exacerbating inflammation and metabolic disorders in this population [74].

##### Dietary carbohydrates

Carbohydrates, particularly nondigestible fibers such as fructooligosaccharides (FOS), galactooligosaccharides (GOS), and inulin, reach the colon intact and serve as substrates for microbial fermentation. This promotes the growth of beneficial bacteria, particularly species belonging to the phyla Bacillota, Bacteroidota, and Actinomycetota [75], such as *F. prausnitzii and members of the genus Bacteroides*, which maintain the gut barrier and exert anti-inflammatory effects [69,76]. Fiber consumption promotes the production of bioactive metabolites such as lactate and SCFAs that have protective functions in aging, including reducing inflammation and stimulating mucin production [76]. In contrast, most dietary starches are digested and absorbed in the small intestine and have minimal effects on the gut microbiota. However, resistant starches reach the colon and support microbial diversity, which is generally associated with better digestive function and overall health.

Dietary fiber plays a key role in preventing and managing various aging-related conditions. However, institutional diets are often low in fiber, contributing to a less diverse and more proinflammatory microbiota. Supplementation with prebiotic fibers such as FOS and inulin has been shown to improve stool frequency and reduce constipation in elderly [77]; this effect is particularly relevant in frail individuals as constipation has been linked to higher frailty levels [78].

To a large extent, the availability of carbohydrates in the colon determines microbial composition, but the effect depends on the amount and type of carbohydrate being consumed. Simple sugars, such as glucose and sucrose, have been associated with an increase in proinflammatory bacteria (*Enterobacteriaceae*) and a decrease in beneficial taxa, such as *Lachnospiraceae* [79]. Preliminary studies also suggest that specific carbohydrates, such as L-arabinose, reduce inflammatory markers and improve the *Bacillota:Bacteroidota* ratio [80].

#### Micronutrients and other specific dietary components

Aging is frequently associated with micronutrient deficiencies due to reduced dietary intake, impaired intestinal absorption, and the use of multiple medications. These deficiencies can exacerbate chronic diseases and frailty in elderly. Moreover, interaction of these micronutrients with the gut microbiota is an emerging area of research, with potential implications for promoting healthy aging [81]. The relevance of these components for institutionalized individuals is summarized in Table 1. Notably, the last column presents the recommendations of the European Society for Clinical Nutrition and Metabolism [82], providing a practical reference for assessing the importance, deficiency risk, and supplementation criteria of each nutrient in the context of institutional care.

**TABLE 1:** Effects of vitamins, minerals, and polyphenols on immunity, gut microbiota, and other geriatric factors in institutionalized elderly.

Micronutrient	Key immune functions	Effects on infections	Interaction with gut microbiota	Evidence in sarcopenia or cognitive function	Relevance in institutionalized elderly	ESPEN recommendations Volkert et al., 2022 [82]
Vitamin C	Antioxidant; supports chemotaxis and phagocytosis; stimulates Ig production	Moderate reduction in cold duration; possible effect in COVID-19	Modulates intestinal oxidative stress, promoting a balanced microbial environment	Possible cognitive improvement; neurovascular protection; associated with skeletal muscle mass	Common deficiency; possible improvement with supplementation	Recommended as antioxidant. Commonly deficient.
Vitamin D	Induces antimicrobial peptides; promotes immune tolerance	Reduces respiratory infections and COVID-19 severity	Increases microbial richness; stimulates beneficial bacteria such as *Akkermansia*	Mild cognitive improvement; less functional decline; deficiency linked to muscle decline	High prevalence of deficiency; relevant for immunity and microbiota	Recommended. High prevalence of deficiency; solid evidence.
Vitamin E	Antioxidant; regulates T and NK lymphocytes; modulates inflammation	Better vaccine response; lower incidence of pneumonia	Reduces intestinal oxidative stress; may support stable microbial communities	Possible role in neuroprotection; protection against muscle aging	Low intake common in institutional diets	Recommended with limited evidence; useful antioxidant.
B-group vitamins (B6, B9, B12)	Involved in immune cell functioning and proliferation, and Treg maintenance	Associated with lower susceptibility to infections	Promote *Lactobacillus* and *Bifidobacterium*; some species synthesize them	Deficiency linked to cognitive decline and anemia; involved in physical functioning	Highly relevant in vegan diets; deficiencies common in institutionalized elderly	Highly relevant. Frequent deficiencies in institutionalized elderly.
Zinc	Regulations of intracellular signaling pathways, essential for T/B lymphocytes, phagocytosis, and NK activity	Supplementation decreases the incidence of infections in the elderly; antiviral properties	Promotes pro-/anti-inflammatory balance; regulates *Enterobacteriaceae*	Linked to better immune-muscle function; possibly linked to muscle strength	Common deficiency; immune function improves with supplementation	Essential. Frequently deficient. Improves with supplementation.
Selenium	Involved in antioxidant activity and part of immunoregulatory selenoproteins	Could reduce respiratory infections	Influences intestinal redox state; potential protective effect against dysbiosis	Associated with cognitive function and healthy longevity; low levels associated with worse sarcopenic outcomes	Low intake in many European regions	Important for immune function. Risk of deficiency in the elderly.
Magnesium	Enzyme cofactor for immunoglobulin synthesis; modulates immune signaling and inflammation	Reduces inflammatory markers	Improves microbial composition; supports SCFA production	Possibly associated with lower risk of cognitive decline; linked to muscle health and physical performance in elderly	Supplementation may be beneficial in oxidative stress scenarios	Worth considering due to its role in metabolism and inflammation.
Iron	ROS production by the Fenton reaction; necessary for cell proliferation	Excess increases infection risk; deficiency causes anemia	Excessive supplementation may increase *Enterobacteriaceae* and dysbiosis	Deficiency linked to fatigue, and reduced cognitive and muscle performance	Risk of dysbiosis due to uncontrolled supplementation	Monitor dosage. Risk of dysbiosis with excessive supplementation.
Calcium	Second messenger in the activation of immune cells	Indirect role in mucosal immunity	Absorption promoted by SCFAs; supports *Bacteroides* and *Faecalibacterium*	Prevents bone loss and sarcopenia	Institutional diets may be deficient	Relevant for bone and muscle mass. Risk of deficiency.
Polyphenols	Modulate inflammation and oxidative stress	Protective effect against chronic infections	Prebiotic effect	Improved cognitive function and cardiovascular health	Emerging evidence for supplementation in nursing homes	Emerging evidence. Potential benefit on microbiota and cognition.

Abbreviations: ESPEN, the European Society for Clinical Nutrition and Metabolism; NK, natural killer; ROS, reactive oxygen species; SCFA, short-chain fatty acid; Treg, regulatory T cell.

##### Vitamins

Among micronutrients, vitamins play a significant role in modulating the gut microbiota. Vitamin D has been associated with increased microbial richness and the promotion of beneficial bacteria such as *A. muciniphila* [83]. B vitamins are essential for the synthesis of key bacterial metabolites, and they promote the growth of *Bifidobacterium* and *Lactobacillus* [84]. Notably, some strains within these genera are also capable of synthesizing B vitamins themselves. However, deficiencies in these vitamins are common among elderly and have been associated with reduced microbial diversity [85]. Vitamins E and C—both potent antioxidants—indirectly modulate the gut microbiota composition by reducing oxidative stress in the intestinal environment, thereby helping preserve microbial balance [86].

##### Minerals

Although less studied, certain minerals can have direct and indirect effects on gut microbial communities. For example, calcium absorption is enhanced by the production of SCFAs and contributes to a gut environment favorable to beneficial bacteria such as *Bacteroides* and *Faecalibacterium*. Magnesium has been shown to influence microbial composition and reduce inflammatory markers [87], whereas zinc and selenium have been associated with a boosting effect on intestinal immunity and help regulate the balance between pro- and anti-inflammatory bacteria [81]. Conversely, excessive iron supplementation has been associated with the overgrowth of pathogenic taxa, including *Enterobacteriaceae* and *E. coli*, which may increase risk of intestinal inflammation [88].

##### Polyphenols and other bioactive compounds

In addition to macro- and micronutrients, other dietary components such as polyphenols have significant effects on the gut microbiome. Polyphenols are bioactive compounds found in plant-based foods and are widely recognized for their protective roles against age-related conditions such as neurodegeneration, cancer, and cardiovascular diseases (CVDs) [89,90]. These benefits are largely mediated through the bidirectional interaction of polyphenols and the gut microbiota. On the one hand, gut microbes metabolize polyphenols into smaller, more bioavailable compounds with enhanced biological activity. On the other hand, polyphenols modulate microbial composition and function, exerting prebiotic-like effects [91]. Despite this potential, most evidence on the effects of polyphenols on the microbiota and health comes from animal models or *in vitro* studies. Human trials and gut biomarkers, particularly those focused on elderly, remain scarce [55,92,93]. Nonetheless, recent findings suggest that polyphenol supplementation in institutionalized elderly individuals improves intestinal permeability markers, reduce blood pressure, and increase the abundance of beneficial taxa such as *Ruminococcaceae* [94]. Furthermore, in elderly, higher daily polyphenol intake (equivalent to ∼178 g fresh weight of blueberries) has been associated with better cognitive performance and a lower risk of cardiovascular and neurodegenerative diseases [95].

## Geriatric Care Factors Affecting Microbiota and Nutrition

**FIGURE 2:**
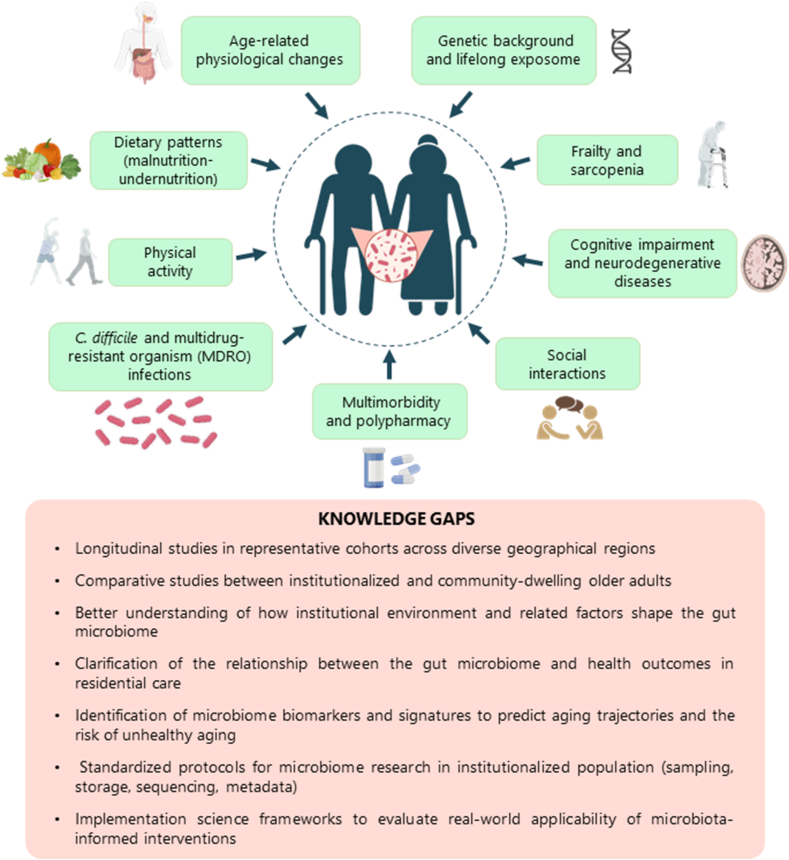
Multidimensional and interacting factors shaping the gut microbiota in institutionalized elderly—including biological (e.g., aging-related physiological changes, genetic background), clinical (e.g., frailty, neurodegeneration, polypharmacy), and environmental (e.g., dietary patterns, social interactions, mobility) dimensions. Despite growing interest in microbiota-targeted strategies, several key knowledge gaps persist that limit translation into effective and scalable interventions in long-term care settings.

In addition to diet, the composition and function of the gut microbiota can be influenced by numerous geriatric conditions and age-related changes, including physiological changes, multimorbidity, chronic inflammation, polypharmacy, infections, cognitive impairment, frailty, and psychosocial factors (Figure 2). In institutionalized elderly, these factors often converge, creating a distinct biological and environmental context that affects both the structure and functionality of the gut ecosystem.

In a long-term care setting, research has primarily focused on specific dimensions such as antibiotic use, colonization by multidrug-resistant organisms (MDROs), malnutrition, frailty, and chronic diseases. These conditions not only have a direct impact on the gut microbiota but can also interact with dietary patterns, thereby influencing nutritional status and health outcomes in a bidirectional and synergistic manner. Table 2 provides an overview of the most relevant human studies conducted to date that investigated the intestinal microbiome among institutionalized elderly populations. It summarizes the study designs, objectives, and principal findings of these studies, offering a comprehensive snapshot of how various geriatric and clinical parameters are related to gut microbiota composition, diversity, and resilience. Collectively, these studies underscore the need for multidisciplinary and context-sensitive approaches to understand microbiota-related dynamics among nursing-home residents.

**TABLE 2:** Overview of human studies exploring the gut microbiome in institutionalized elderly.

Reference	Study design	Objective	Characteristics	Results
Kitamura et al., 2024 [96]	Observational, cross-sectional and within-subject; *n* = 27 (oral samples) and *n* = 101 (stool samples); Japan	To explore the effects of antimicrobial treatment on the human microbiome and resistome.	47 treatment and 34 without treatment. Shotgun metagenome sequencing of oral and stool samples.	Cross-sectional analysis revealed that *Methanobrevibacter* and total abundance of antimicrobial resistance genes were significantly increased in stool samples after antibiotic treatment. Within-subject comparison suggested that the effect of the prescription of a single antimicrobial drug in usual clinical treatment on the gut microbiota is likely to be smaller than previously thought.
Wang et al., 2023 [97]	Observational, retrospective study; *n* = 245; United States	To identify resident characteristics associated with VRE-colonized residents contaminating their environment.	Demographic and clinical data collection. Follow-up for ≤6 mo. 16S rRNA gene sequencing on perirectal swabs.	At baseline, VRE colonization was present in 20% of participants, with environmental surface contamination near 73% of these patients. Microbiota composition was associated with antibiotic receipt within the past 30 d and VRE colonization status but not environmental contamination among VRE-colonized participants.
Liu et al., 2022 [98]	Interventional, randomized-controlled trial; *n* = 7; United States. Mean age = 82.2 ± 8.5 y, 62.7 % female	To determine the feasibility and safety of aFMT in NHs.	3 controls and 4 fecal transplanted subjects. Stool sample collection, 16S rRNA gene sequencing analysis, and sample preparation for aFMT.	aFMT has limited feasibility in a NH population due to logistic and technical challenges but is likely safe.
Ishida et al., 2022 [99]	Observational, preliminary cross-sectional study; *n* = 23; Japan	To study the relationship between sarcopenia diagnostic parameters and the diversity of gut microbiota in elderly living in NHs	23 participants who were clinically stable. Stool sample collection and 16S rRNA gene sequencing analysis.	The microbiota of frail and sarcopenic participants tended to be less diverse, although not significantly.
Ducarmon et al., 2021 [100]	Observational, prospective, longitudinal cohort study; *n* = 27; Netherlands	To investigate the role of the gut microbiota in providing colonization resistance against MDROs.	Four time points with a 2-mo interval. Stool sample collection and microbiota sequencing analysis.	Antibiotic use in the previous 2 mo and hospital admittance in the previous year were associated with MDRO colonization. No differences in alpha or beta diversity between groups. *Dorea*, *Atopobiaceae*, and *Lachnospiraceae* ND3007 groups were more abundant in residents never colonized with an MDRO throughout the 6-mo study. A high abundance of *Bifidobacterium* was observed in several residents.
Haran et al., 2021 [31]	Observational, longitudinal study; *n* = 166; United States	To define the factors that have the greatest association with NH residents’ gut microbiota and explore patterns of dysbiosis and compositional changes in gut microbiota over time in this environment.	15 residents followed for 1 y. Stool sample collection and nutritional and frailty assessment. Shotgun sequencing analysis.	Medications, particularly psychoactive and antihypertensive medications, had the greatest effect on the microbiota. Age and frailty also contributed and were associated with increased and decreased diversity, respectively. The microbiota of residents who had lived in the NH for >1 y was enriched in inflammatory and pathogenic species and reduced in anti-inflammatory and symbiotic species.
Haran et al., 2021 [101]	Observational, prospective cohort study; *n* = 167; United States	To explore the prevalence of *C. difficile* in NH elders over time and to determine whether the microbiome or other clinical factors are associated with *C. difficile* colonization	Stool and data collection. Shotgun sequencing analysis.	Overall, 46.7 % had *C. difficile-*positive samples. Samples positive for *C. difficile* had increased abundance of pathogenic or inflammatory-associated bacterial taxa and lower abundance of taxa with anti-inflammatory or symbiotic properties.
Wang et al., 2020 [102]	Observational, prospective, longitudinal cohort study; *n* = 61; United States	To determine if antibiotic use at health care facilities could impact patient risk of ARO acquisition in NHs.	14-d enrollment. Perirectal swab collection and 16s rRNA gene sequencing analysis.	Recent exposure to high-risk antibiotics was associated with risk of ARO acquisition. Patients with elevated *Enterococcus* abundance were at a higher risk of enteric ARO acquisition.
Le Bastard et al., 2020 [103]	Observational, retrospective study; *n* = 144; France	To identify specific bacteria and functional pathways, and genes characterizing the gut microbiome of NH residents carrying extended ESBL-E.	Stool sample collection and shotgun sequencing analysis.	Some residents were ESBL-E carriers. Their microbiome was depleted in butyrate-producing species and enriched in succinate-producing species, and pathways involved in intracellular pH homeostasis were affected.
Haran et al., 2019 [104]	Observational, longitudinal study; *n* = 108; United States	To explore the microbiome composition of NH elders with AD, no dementia, or other dementia types to understand how specific intestinal bacterial taxa associate with AD and the extent to which such taxa alter the balance of intestinal epithelial homeostasis.	Follow-up for 5 mo. Metagenomic sequencing and in vitro T84 intestinal epithelial cell functional assays for P-glycoprotein expression.	Some clinical parameters and numerous microbial taxa and functional genes act as predictors of AD dementia in comparison to elders without dementia or with other dementia types. Stool samples from elders with AD can induce lower P-glycoprotein expression levels in vitro*.* The microbiome of persons with AD shows a lower proportion and prevalence of butyrate-producing bacteria and higher abundances of proinflammatory taxa.
Araos et al., 2019 [105]	Observational, retrospective study; *n* = 82; United States	To characterize the fecal microbiome of geriatric residents and compare their resistome with that of healthy young individuals.	82 elderly compared with healthy adults from the Human Microbiome Project. Shotgun and 16S rRNA gene sequencing.	The microbiome of the elderly institutionalized population is substantially dysbiotic, and their resistome is composed of numerous antibiotic resistance genes. Differences in bacterial taxa were observed according to whether or not MDROs were acquired.
Haran et al., 2018 [106]	Observational, prospective, longitudinal cohort study; *n* = 23; United States	To explore the associations of NH environment, frailty, nutritional status, and residents’ age with microbiome composition and potential metabolic function.	Resident assessment using the mini nutritional assessment tool and clinical frailty scale. Stool samples collection and 16S rRNA gene sequencing analysis.	The abundance of some important microbiota-encoded genes and pathways declined with age. Residents had lower abundances of butyrate-producing organisms with increasing frailty. Butyrate-producing organisms declined and dysbiotic bacterial species increased with malnutrition. The microbiome of residents living in proximity shared similar species.
Araos et al., 2018 [107]	Observational, retrospective study; *n* = 28; United States	To assess the diversity and composition of the gut microbiota among residents with advanced dementia colonized with *C. difficile.*	21 controls (*Clostridium difficile-*negative) and 7 cases (*C. difficile*-positive). Stool sample collection and 16S rRNA gene sequencing analysis.	Alpha and beta-diversity were similar between cases and controls. Cases showed a higher relative abundance of *Akkermansia* spp., *Dermabacter* spp., *Romboutsia* spp., *Meiothermus* spp., *Peptoclostridium* spp., and *Ruminococcaceae* UGC 009.
D’Agata et al., 2018 [108]	Observational, nested case-control study; *n* = 137; United States	To identify risk factors associated with MDRO acquisition among residents not exposed to antimicrobials.	Rectal and nasal samples collected.	Overall, 57 MDRO isolates were acquired among cases, including multidrug-resistant gram-negative bacteria, methicillin-resistant *Staphylococcus aureus,* and vancomycin-resistant *enterococci.*
Jeffery et al., 2016 [32]	Observational study; *n* = 371; Ireland	To refine the understanding of diet–microbiota associations and differential taxon abundance.	Four groups: community-dwelling (community), attending outpatient day hospitals (day hospital), in short-term rehabilitation care (rehab, <6 wk), or in long-term care facilities (long-stay). Clinical and nutritional data collected. Stool sample collection and 16S rRNA gene sequencing analysis.	Distinctive microbiota configurations associated with aging in both community and long-stay residential care elderly subjects were identified. Changes in long-stay bacterial populations represent the loss of the health-associated and youth-associated microbiota components and gain of an elderly associated microbiota. Community-associated microbiota showed more loss but also more recovery after antibiotic treatment.
Rodriguez et al., 2016 [109]	Observational, longitudinal study; *n* = 23; Belgium	To evaluate and follow the prevalence of *C. difficile* in NH residents.	Weekly evaluation for 4 mo. Stool sample collection and 16S rRNA gene sequencing analysis.	Overall, 30.4% of residents were positive for *C. difficile*. Each resident had their own bacterial imprint, which was stable during the study. *C. difficile*-positive individuals showed significant changes in the relative abundance of a few bacterial populations and decreased *Akkermansia.*
Gillespie et al., 2015 [110]	Observational, prospective cohort study; *n* = 279; United Kingdom	To study the frequency and risks for antibiotic prescribing and AAD in care home residents.	12-mo study.	The incidence of antibiotic prescriptions was 2.16 prescriptions per resident year. AAD was more likely in residents who were prescribed co-amoxiclav or routinely used incontinence pads.
Claesson et al., 2012 [30]	Observational; *n* = 178; Ireland	To test the hypothesis that variation in the intestinal microbiota of older subjects has an impact on immunosenescence and frailty across the community.	83 community dwelling; 20 outpatient day hospital, 15 short-term rehabilitation hospital care; 60 long-term residential care.	The fecal microbiota composition correlated with residence location. Clustering by diet separated them by the same residence location and microbiota groupings. The separation of microbiota composition significantly correlated with measures of frailty, comorbidity, nutritional status, markers of inflammation, and with metabolites in fecal water. The individual microbiota of people in long-stay care were significantly less diverse than those of community dwellers. Loss of community-associated microbiota correlated with increased frailty.

Abbreviations: AAD, antibiotic-associated diarrhea; AD, Alzheimer’s disease; aFMT, autologous fecal microbiota transplant; ARO, antibiotic-resistant organism; ESBL-E, β-lactamase-producing *Enterobacteriaceae*; MDRO, multidrug-resistant organism; NH, nursing home; VRE, vancomycin-resistant enterococci.

In the following subsections, we review the current evidence on the main factors influencing the gut microbiota in institutionalized elderly, focusing on immune system alterations and infections, frailty and sarcopenia, cognitive decline and neurological disorders, and the complex effects of multimorbidity and polypharmacy. We also briefly discuss their potential interactions with diet and implications in gut-related health outcomes, highlighting key research gaps outlined in Figure 2.

### Immunological changes and infections in the geriatric population

Aging is accompanied by chronic stimulation of the immune system, leading to immunosenescence and a state of low-grade inflammation known as inflammaging. These immune alterations impair the innate and adaptive responses, increasing risk of noncommunicable diseases such as frailty, cancer, and neurodegeneration [111,112]. In particular, the decline in naïve T cells, thymic involution, and increased senescent natural killer and T cells can reduce immune tolerance and promote chronic inflammation. This change, together with the loss of CD28^+^ T cells, reduces the immune tolerance toward the microbiome, favoring chronic inflammation [113,114].

In institutionalized elderly, immunosenescence is exacerbated by frailty and poor nutritional status, contributing to alterations in the gut microbiota. Conversely, age-associated dysbiosis may worsen immune dysfunction, creating a vicious cycle of inflammation and microbial imbalance [115,116]. Diet plays a central role in this crosstalk: nutrients modulate epithelial barrier integrity, gut-associated lymphoid tissue (GALT), and microbiota-derived metabolites such as SCFAs, which impact both local and systemic immunity [117,118].

Key micronutrients, such as vitamins C, D, and E; zinc; and selenium, support immune function and may partially reverse age-related deficits, although responses vary among individuals. Among the dietary factors involved, these micronutrients play critical roles in supporting various immune functions. Evidence suggests that supplementation with these micronutrients may reverse immune deficits in elderly, particularly in those with pre-existing deficiencies. However, findings across studies remain inconsistent, potentially because of high interindividual variability in immune markers and the modest effect sizes observed with nutritional interventions [119].

Nutrients can modulate immune function through both systemic and local mechanisms. Once absorbed, they act on immune-related sites such as the bone marrow, thymus, and secondary lymphoid organs. Additionally, certain dietary components exert local effects in the gut without systemic absorption by enhancing epithelial barrier integrity and modulating GALT [117]. These components also shape gut microbiota composition, influencing the immune–microbiota crosstalk through the production of metabolites such as SCFAs, which have both local and systemic immunomodulatory effects [117,120]. Furthermore, GALT-primed immune cells can migrate to distal sites, including the respiratory tract, allowing gut-originated signals to impact extraintestinal immunity [117].

Immunosenescence, multimorbidity, and shared living conditions in nursing homes significantly increase risk of infections, including urinary, respiratory, skin, and gastrointestinal infections. These are often diagnosed late because patients present with nonspecific symptoms, blunted febrile responses, and sampling difficulties [121], contributing to antibiotic overuse and the emergence of MDROs and *C. difficile* infection (CDI) [122]. The institutionalized elderly often act as reservoirs of MDROs because of severe dysbiosis. In residents with dementia, the gut microbiota was highly altered and rich in antibiotic resistance genes, suggesting that microbial composition influences MDRO colonization risk [105].

CDI is highly prevalent in nursing homes, with colonization rates ranging from 20% to 50%, which is far higher than that in the general population [101]. Age-related dysbiosis, along with antibiotics and proton pump inhibitors, increases susceptibility [123]. Individuals with CDI show decreased prevalence of butyrate-producing bacteria and increased prevalence of proinflammatory species [107,124], including *Bacteroides fragilis*, *Eggerthella lenta*, *E. coli*, *Shigella* spp., and Pseudomonadota species, as well as *Anaerostipes caccae*, *Phocaeicola vulgatus* (formerly known as *B. vulgatus*), and *R. gnavus* [101]; other additional changes are also observed, such as increased *Blautia* and decreased *Akkermansia* [109].

The clinical spectrum of CDI ranges from asymptomatic carriage to severe colitis and death. Microbiota-mediated resistance depends on gut metabolic activity, particularly bile acids that regulate *C. difficile* germination and growth [125]. Antibiotics disrupt the balance between primary and secondary bile acids, increasing disease risk. These dynamics support the therapeutic use of fecal microbiota transplantation, which has been tested in pilot studies in nursing homes [98].

Diet also modulates CDI risk. Diets high in fat and protein exacerbate infection in animal models, whereas microbiota-accessible carbohydrates increase SCFAs that inhibit *C. difficile* [126]. Although zinc deficiency is common in the elderly, excess zinc intake may promote dysbiosis and infection [127]. Nonsteroidal anti-inflammatory drugs may further worsen outcomes by impairing microbiota balance, immune responses, and gut barrier integrity [125].

### Frailty and sarcopenia

Frailty is a multifactorial geriatric syndrome marked by decreased physiological reserves and reduced resilience to stressors, whereas sarcopenia involves the progressive loss of muscle mass and function [128]. Both conditions are highly prevalent among individuals living in nursing homes [129,130]. The concept of cognitive frailty, which combines physical frailty with cognitive impairment in the absence of dementia, has gained recognition as a multidimensional syndrome that may precede neurodegenerative disease [131].

Although tools such as Fried’s Phenotype and Rockwood’s Index exist, diagnostic criteria for frailty are not yet standardized [131]. Its prevalence is estimated at 50% in nursing-home residents and ∼10% in community-dwelling elders [130]. Frailty increases disability and risk of institutionalization [132], and sarcopenia, which is also more prevalent in nursing homes, is a strong predictor of mortality [133]. In these settings, sarcopenia affects ∼51% of women and ∼31% of men.

Frailty and sarcopenia overlap in their physical manifestations and share malnutrition as a key driver [128,129]. Both undernutrition and obesity contribute to frailty risk in elderly living in the community [128], and age-related anorexia promotes chronic malnutrition, functional decline, and sarcopenia [134]. The co-occurrence of malnutrition and frailty is linked to increased hospitalization, morbidity, and mortality [135].

The gut microbiota appears to play a role in these conditions. Frailty has been associated with a reduction in butyrate-producing taxa (*Faecalibacterium*, *Roseburia*) and enrichment of pathobionts (*Eggerthella*, *B. fragilis*) [136]. Sarcopenia correlates with lower microbial diversity and a decline in beneficial microbes, supporting the concept of a gut–muscle axis [[137], [138], [139]]. In institutionalized populations, frailty has been linked to reduced diversity, depletion of beneficial microbes, and increased dysbiosis-associated species [31,106], along with metabolic signatures such as enhanced LPS and peptidoglycan biosynthesis and altered sphingolipid metabolism. Frailty and malnutrition also coincide with elevated *R. gnavus* and reductions in *Lachnospiraceae* and *Ruminococcaceae* [106], suggesting these to be microbial markers of vulnerability.

Although clinical trials have yielded mixed results because of variability and heterogeneity, comprehensive dietary strategies are increasingly recommended. These emphasize high-quality proteins, essential micronutrients, anti-inflammatory components, and chrononutrition principles to optimize metabolic responses. Tailoring interventions to individual profiles, including comorbidities and medication use, is especially critical in institutional settings where nutritional risk is elevated and response to standard strategies is often suboptimal [140].

### Cognitive decline and neurological disorders

Aging is accompanied by cognitive changes ranging from mild impairment to severe neurodegenerative conditions such as dementia. Although normal cognitive decline may not affect daily activities [141], aging is a major risk factor for diseases such as Alzheimer’s disease (AD) and Parkinson’s disease that involves interaction between genetic and environmental factors [[142], [143], [144]].

Cognitive impairment, which often coexists with frailty, is a major cause of institutionalization. Its prevalence in nursing homes (∼21.2%) slightly exceeds that in the community (∼17.3%) [145,146]. Moreover, institutional living may accelerate cognitive decline and promote depression, which often co-occurs with cognitive impairment, and increases risk of dementia progression [147,148].

Emerging evidence highlights the role of the gut microbiome in brain function and neurodegeneration through the gut–brain axis, which integrates immune, metabolic, and neuroendocrine signals between the gut and the central nervous system [7,149,150]. Age-related chronic inflammation also promotes neuroinflammation and cognitive decline in close interaction with gut dysbiosis.

Most studies on the microbiome and cognition focus on AD in noninstitutionalized populations. A recent meta-analysis revealed that microbial alterations occur even in the early stages of AD [146]. In nursing homes, only the study by Shoubridge et al. [151] explored this link, reporting that individuals with severe cognitive impairment exhibit higher levels of *Methanobrevibacter smithii*, reduced levels of *Bacteroides uniformis*, and diminished potential for SCFA and neurotransmitter synthesis along with enhanced methanogenesis.

Despite growing interest in the role of the gut–brain axis in psychiatric conditions, institutionalized elderly remain underrepresented in this research. Nutritional deficits are also common; many nursing-home residents fail to consume the recommended intake of vitamins A, B (including vitamin B_12_), D, and E and zinc, which are essential for immune and cognitive function [152]. In this sense, a recent report from The Lancet Commission on Dementia Prevention, Intervention, and Care estimated that ≤45% of dementia cases could be prevented through the modification of risk factors, with nutrition increasingly recognized as one of them [153]. Particularly, vitamin B12 deficiency has been linked to increased frailty and reduced functional status in elderly [154].

Polyphenol intake is also inadequate among nursing-home residents. In the MaPLE ('Microbiome mAnipulation through Polyphenols for managing Leakiness in the Elderly') study, actual polyphenol consumption was 15% lower than planned because of reduced intake, despite menus comprising ∼770 mg/d [155]. This shortfall may negatively impact microbiota composition, intestinal barrier function, and metabolic health.

Souvenaid® is a nutritional supplement that is specifically formulated to improve synapse formation and function. It contains a combination of key nutrients, including ω-3 PUFAs (EPA and DHA), B vitamins, antioxidants, and uridine monophosphate [156]. Several clinical trials in patients with mild AD have shown that Souvenaid could improve memory and help preserve brain network organization [[156], [157], [158]]. Despite these promising effects on cognitive function, its potential impact on the gut microbiota and the implications of this interaction for brain health remain unexplored.

### Multimorbidity, polypharmacy, and their impact on the gut microbiome in institutionalized elderly

Aging is a key risk factor for chronic diseases such as CVD, diabetes, neurodegenerative conditions, cancer, and musculoskeletal disorders [159,160]; many of these, such as diabetes and hip fractures, are linked to institutionalization due to functional decline [161].

Multimorbidity, commonly defined as the coexistence of ≥2 chronic diseases, affects >65% of people aged 65–84 y and >80% of those aged >85 y [162]. It is associated with higher mortality, hospitalizations, disability, depression, and dependence on long-term care [162,163]. Despite increasing interest in microbiota–disease interactions, data on institutionalized populations remain scarce (Table 2).

Gut microbiota alterations have been linked to various chronic diseases. In CVD, microbial shifts and altered metabolite profiles (e.g., imidazole propionate) may influence disease progression [164,165]. For example, hypertension has been linked to a reduction in SCFA-producing microbes such as *Faecalibacterium* and *Roseburia*, along with an increase in proinflammatory taxa, such as *Klebsiella* and *Clostridium* [166]. Moreover, the microbial metabolite imidazole propionate, derived from histidine, has been associated with poor survival outcomes in patients with heart failure [167]. Other conditions, such as dyslipidemia and osteoporosis, also involve microbiota disturbances through mechanisms such as SCFA production, bile acid metabolism, or bone turnover modulation [[168], [169], [170]].

Multimorbidity often leads to polypharmacy (≥5 drugs), affecting ≤91% of institutionalized adults—far higher than the 27% to 59% in the community [171,172]. Although necessary in many cases, polypharmacy increases risk of adverse drug events, especially in frail and institutionalized individuals, where ≤91% of residents are affected [173]. Excessive polypharmacy (≥10 drugs) and inappropriate prescriptions are particularly frequent after admission to residential care [174], often driven by high rates of cognitive and psychiatric conditions [175].

The interaction between polypharmacy and the gut microbiota is an emerging area of study. Beyond antibiotics, many medications modulate microbiota composition and function. *In vitro*, 24% of 1000 tested drugs inhibited the growth of ≥1 gut bacterial strain [176], highlighting the broad potential for unintended microbial disruption. For example, proton pump inhibitors are associated with overgrowth of *Enterobacteriaceae*, *Lactobacillaceae*, and oral-origin taxa, with reductions in *Ruminococcaceae* and *Bifidobacteriaceae* [177,178]. Functional consequences included altered lipid and NAD metabolism [179]. Metformin increases *E. coli* and *Akkermansia*, although its effects vary across cohorts [[180], [181], [182]].

The role of the microbiota in drug metabolism—affecting not only pharmacokinetics but also toxicity and efficacy—is also increasingly recognized as critical for drug development [[183], [184], [185]]. However, data on the institutionalized elderly remain scarce. One notable study found that medication use alone could predict ≤60% of the gut microbiome composition in nursing-home residents, with psychotropic and antihypertensive drugs having the greatest impact [31]. This underscores the urgent need for further research in this vulnerable population to better understand the implications of polypharmacy on gut microbiota and overall health.

Finally, the interaction between drugs, diet, and microbiota is a promising area. A study in Swedish adults (*n* = 2223) linked nonantibiotic medications and polypharmacy to major microbial shifts [186]. Although diet was not assessed, other research supports the role of specific dietary components in buffering drug-induced dysbiosis. Resources such as FooDrugs and Diet–Drug Interactions Database [187,188] systematize knowledge on food–drug interactions, paving the way for personalized, microbiome-informed interventions.

## Reasonable Dietary and Multimodal Recommendations to Support Healthy Aging in Institutionalized Residents

### Toward more individualized diets in nursing homes: from functional adaptation to microbiota-informed strategies

Institutional food provision often follows standardized menus based on general dietary guidelines, yet these rarely account for the heterogeneity of elderly in terms of nutritional status, comorbidities, or microbiome profiles. However, personalizing diets in nursing homes is crucial to address the interplay between malnutrition, chronic diseases, and gut microbiota alterations.

Dietary approaches for institutionalized elderly need to address functional limitations, including dysphagia, impaired digestion, or appetite loss, as well as the complex, bidirectional interactions between diet and the gut microbiota. On a practical level, strategies such as introducing texture-modified meals, energy-dense small portions, and sensory-enhanced foods have demonstrated efficacy in improving food intake and satisfaction among residents with feeding difficulties. Clinical evidence supports the benefits of these adaptations in enhancing nutritional status and quality of life in long-term care settings [189]. These measures are further supported by implementation frameworks that emphasize the importance of staff involvement, mealtime experience, and alignment with best practice guidelines [190].

However, moving beyond functional adaptation toward truly individualized dietary patterns remains a major challenge in nursing homes. Standardized menus often fail to accommodate individual needs, and implementation depends on broader health system and organizational factors [191]. Moreover, interindividual variability in dietary responses is shaped by both extrinsic (food composition and processing) and intrinsic factors (age, sex, comorbidities, polypharmacy, and genetic background), with the gut microbiota playing a central role [192,193]. Even beyond traditional nutritional targets, growing evidence links interindividual differences in glycemic control, inflammatory tone, and nutrient metabolism to microbiota composition and function, suggesting that conditions such as osteoporosis also benefit from microbiota-informed dietary modulation [194,195].

Therefore, dietary recommendations in institutional settings should prioritize diversity, sufficient protein and micronutrient intake, inclusion of fiber-rich and fermented foods, and alignment with microbiota-supporting dietary patterns. In addition to macronutrient targets, interventions must also adapt to practical considerations, including assistance during meals, flexibility in mealtime schedules, and continuous reassessment of dietary needs. These elements are essential in long-term care settings, where residents’ health status often changes rapidly and heterogeneity is the norm. Along with nutrition, medication management must be integrated into personalized care. Thus, considering drug–microbiota–diet interrelations is crucial when designing individualized nutrition plans. Integrating dietary and pharmacological management through a microbiome-informed lens could enhance the overall impact of interventions [191].

### Digital technologies and artificial intelligence for personalized nutrition

To support and scale personalized dietary approaches in nursing homes, there is growing interest in incorporating digital technologies and artificial intelligence (AI) in the context of long-term care. These tools can complement clinical and nutritional assessments by enabling more accurate, dynamic, and individualized nutritional strategies, even in populations with high clinical complexity.

Machine learning models integrating microbiota, metabolomic, and clinical data have demonstrated the ability to predict postprandial glycemic responses and guide more effective, individualized dietary strategies [194,196]. For example, the ZOE PREDICT program uses multiomic data to generate personalized diet quality scores, resulting in improved cardiometabolic health [196]. Similarly, the Life’s Essential 8 framework has been applied to large aging cohorts to stratify risk of cardiovascular disease and provide preventive recommendations [197]. In parallel, microbial community-scale metabolic models have emerged as a promising tool for designing personalized prebiotic, probiotic, or dietary interventions aimed at optimizing specific metabolic outputs or ecological behaviors in the gut [198]. To date, these models have been successfully used to predict individual-specific production of SCFAs in response to targeted prebiotic or probiotic interventions, with outcomes correlating to cardiometabolic and immunologic health markers [199].

In addition to these research-driven initiatives, more applied digital solutions are emerging for use in care settings. For example, AI-powered natural language processing systems have shown promise in detecting malnutrition risk by analyzing unstructured clinical notes from electronic health records [200]. Digital food logs and mobile applications can support the continuous tracking of intake, gastrointestinal symptoms, and food preferences, allowing for real-time dietary adjustments. Some platforms also incorporate recommendation engines that suggest individualized menus based on medical conditions, nutritional needs, and microbiota-informed criteria [196].

Even the partial adoption of such technologies in nursing homes can yield meaningful benefits. Examples of these implementable technologies include digital registries for tracking weight loss, automated alerts for potential nutrient deficiencies, and clinical decision support tools that assist with supplement prescription and monitoring. One illustrative case is a guideline-based decision support system that is implemented in French nursing homes which improved the management of malnutrition by aligning recommendations with clinical guidelines [201].

In addition to individual tailoring, these tools enhance interdisciplinary coordination by enabling more effective communication among dietitians, physicians, caregivers, and pharmacists. This is particularly relevant in settings with high clinical complexity, where polypharmacy and multimorbidity are common and may impact nutrient absorption or interact with the microbiota. Digital platforms that consolidate relevant data can help align dietary and pharmacological strategies to maximize intervention efficacy.

Despite the promise of these interventions, ethical and practical challenges remain, including data privacy, digital literacy, infrastructure limitations, and the need for adequately trained staff. Nonetheless, the ongoing digital transformation of nutritional care represents a key opportunity to enhance personalization, adherence, and long-term sustainability of dietary interventions in institutionalized elderly.

### Probiotic supplementation in institutionalized elderly

Building on the potential of digital tools to guide personalized dietary strategies, another emerging avenue involves the use of probiotics and microbiota-targeted supplements to support gut health in institutionalized elderly. Specific probiotic strains have shown potential benefits in managing age-related conditions by modulating immune responses, reducing inflammation, and influencing neuroendocrine and metabolic pathways. Preclinical studies in *C. elegans* and mice models have reported lifespan extension and the development of resistance to oxidative stress and infections with strains like *Lacticaseibacillus gasseri*, *Propionibacterium freudenreichii*, and *A. muciniphila* [202,203]. In humans, certain probiotics, such as *L. reuteri,* have been associated with improved bone health, reduced inflammatory markers in rheumatoid arthritis, and improved gastrointestinal symptoms and cognitive function in individuals with AD or Parkinson’s disease [204,205]. Despite these promising findings, clinical trials remain limited in both number and methodological robustness, and the mechanistic understanding of these effects is still evolving.

In the context of institutionalized elderly, probiotic-based interventions have focused primarily on enhancing immune function and reducing risk of infections, particularly those of the respiratory tract (Table 3). The most commonly studied strains belong to the genera *Bifidobacterium* and *Lactobacillus* (now partially reclassified, e.g., *Lacticaseibacillus rhamnosus* GG), which have shown encouraging results in managing such conditions [226]. Notably, *Lacticaseibacillus casei Shirota* has been associated with reduced fever episodes, decreased incidence of infections, and improved bowel habits in elderly populations [214,220,222]. Additionally, multistrain formulations—mainly combinations of *Bifidobacterium* and *Lactobacillus* species—have shown potential in preventing antibiotic-associated diarrhea and reducing colonization by MDROs [212,213].

**TABLE 3:** Intervention with probiotics in nursing homes.

Reference	Target	Study design	Product	Results
Mozota et al., 2022 [206]	Improve health status	Single-arm, open-label, pre–post study; 4 mo; *n* = 25; Spain	Fermented dairy product with 9.3 log_10_ CFU/product of *Ligilactobacillus salivarius* CECT 30632 (previously known as *L. salivarius* MP101)	*L. salivarius* CECT 30632 may have contributed to improvements in their functional, nutritional, and immunological status without changing the general structure of their nasal and fecal bacteriomes when assessed at the genus level.
Šola et al., 2022 [207]	Evaluate the efficacy and safety on functional constipation	Randomized, double-blind, placebo-controlled, parallel-group, pre–post study; 12 wk; *n* = 60; Croatia	Liquid probiotic containing *Lactobacillus acidophilus* LA3 (1 × 10^9^ CFU/g), *Bifidobacterium animalis* subsp. *lactis* BLC1 (1.5 × 10^9^ CFU/g) and *Lactobacillus casei* BGP93 (2 × 10^9^ CFU/g)	Multistrain probiotic supplementation was found to be efficacious, safe, and well tolerated in the elderly with functional constipation.
Fernández-Ferreiro et al., 2022 [208]	Improve immune response to COVID-19 vaccination	Randomized, double-blind, placebo-controlled, parallel-group, pre–post study; 3 mo; *n* = 200; Spain	Probiotic containing 3 × 10^9^ CFU of *Loigolactobacillus coryniformis* K8	The administration of K8 may enhance the specific immune response against COVID-19 and may improve the COVID-19 vaccine-specific responses in elderly populations.
Mozota et al., 2021 [209]	Improve functional, cognitive, and nutritional status and inflammatory profiles	Single-arm, open-label, pre–post study; 4 mo; *n* = 25; Spain	Fermented dairy product with 9.3 log_10_ CFU/unit with *L. salivarius* MP101	*L. salivarius* MP101 seems to be a promising strain for improving or maintaining health in residents of nursing homes.
Castro-Herrera et al., 2021 [210]	Improve the immune system	Randomized, double-blind, placebo-controlled, parallel-group, pre–post study; ≤12 mo; *n* = 179; United Kingdom	Probiotic containing LGG and BB-12 (1.3 × 10^10^ to 1.6 × 10^10^ CFU/capsule)	Intervention with the combination of LGG plus BB-12 did not have any effect on immune biomarkers, although there was an indication that the probiotics improved the response to seasonal influenza vaccination with significantly higher seroconversion to one strain of the quadrivalent vaccine.
Butler et al., 2020 [211]	Reduce antibiotic administration	Randomized, double-blind, placebo-controlled, parallel-group, longitudinal study; 12 mo; *n* = 195; United Kingdom	Probiotic containing LGG and BB-12 (1.3 × 10^10^ to 1.6 × 10^10^ CFU/capsule)	A daily dose of a probiotic combination of LGG and BB-12 did not significantly reduce antibiotic administration for all-cause infections.
Van Wietmarschen et al., 2020 [212]	Prevent antibiotic-associated diarrhea	Pragmatic participatory evaluation, single-arm, open-label; upon start of antibiotic treatment, ≤1 wk after completing treatment; *n* = 93; Netherlands	Ecologic AAD, with 9 bacterial species at 10^10^ CFU per species: *Bifidobacterium bifidum* W23, *Bifibacterium longum* W51, *Enterococcus faecium* W54, *Lactobacillus acidophilus* W37 and W55, *Lactobacillus paracasei* W20, *Lactobacillus plantarum* W62, *Lactobacillus rhamnosus* W71, and *Lactobacillus salivarius* W24	Successful implementation of probiotics demonstrated the prevention of antibiotic-associated diarrhea in nursing-home residents.
Zollner-Schwetz et al., 2020 [213]	Reduce intestinal and skin colonization by MDRO Gram-negative bacteria	Single-arm, open-label, longitudinal study; 3 mo; *n* = 12; Austria	Omnibiotic10 AAD, with 10 different bacterial strains: *Enterococcus faecium* W54, *Lactobacillus acidophilus* W55 and W37, *Lactobacillus paracasei* W72, *Lactobacillus rhamnosus* W71, *Lactobacillus salivarius* W24, *Lactobacillus plantarum* W62, *Bifidobacterium bifidum* W23, *Bifidobacterium lactis* W18, and *Bifidobacterium longum* W51	A 12-wk course of a multispecies probiotic led to a transient reduction of intestinal colonization by MDROs for 8 wks after the end of treatment.
Kushiro et al., 2019 [214]	Reduce fever	Randomized, double-blind, placebo-controlled, parallel-group, pre–post study; 6 mo; *n* = 880; Japan	Fermented milk containing 1.5 × 10^10^ CFU/bottle of LcS	Continuous intake of the fermented milk could be beneficial for the elderly in terms of suppressing the number of days of detection of fever and the duration of fever.
Fonollá et al., 2019 [215]	Enhance immune response to the influenza vaccine	Randomized, double-blind, placebo-controlled, parallel-group, longitudinal study; 5 mo; *n* = 98; Spain	Probiotic containing 3 × 10^9^ CFU/d of *Lactobacillus coryniformis* K8 CECT5711	The administration of *L. coryniformis* K8 CECT5711 to an elderly population increased the immune response against the influenza vaccine and decreased symptoms associated with respiratory infections.
Yamamoto et al., 2019 [216]	Enhance immunity to influenza virus	Randomized, double-blind, placebo-controlled, parallel-group, longitudinal study; 12 wk; *n* = 96; Japan	Yogurt fermented with the strain *Lactobacillus delbrueckii* spp. *bulgaricus* OLL1073R-1 (1.8 × 10^8^ to 3.5 × 10^8^ CFU/g)	Continuous daily ingestion of yogurt with OLL1073R-1 strain may help prevent infection with influenza A virus subtype H3N2 in elderly subjects with weakened immunity, by increasing the production of influenza A virus subtype H3N2-bound salivary IgA.
Wang et al., 2018 [217]	Prevent respiratory infections	Randomized, double-blind, placebo-controlled, parallel-group, longitudinal study; 6 mo; *n* = 196; Canada	Probiotic containing 10^10^ CFU/capsule of LGG	No significant effects of LGG in reducing influenza and other respiratory virus infections were observed in residents of nursing homes.
Maruyama et al., 2016 [218]	Improve the immune system	Randomized, double-blind, placebo-controlled, parallel-group, pre–post study; 6 wk; *n* = 42; Japan	Jelly containing 10 billion heat-killed *Lactobacillus paracasei* MCC1849	No significant effects of nonviable *L. paracasei* MCC1849 were observed in the elderly.
Nagata et al., 2016 [219]	Normalize bowel movements and improve infection control	Randomized, double-blind, placebo-controlled, parallel-group, longitudinal study; 6 mo; *n* = 72; Japan	Yakult 400, a fermented milk containing 4 × 10^10^ CFU/bottle of LcS	The long-term consumption of LcS-fermented milk may be useful for decreasing the daily risk of infection and improving the quality of life among the residents and staff of facilities for the elderly.
van den Nieuwboer et al., 2015 [220]	Improve bowel habits	Placebo-controlled, parallel-group, longitudinal study; 9 wk; *n* = 44; Netherlands	Fermented milk drink containing minimally 6.5 × 10^9^ CFU/bottle of LcS	A fermented milk containing LcS significantly improves the bowel habits of frail elderly residents in a nursing home.
Van Puyenbroeck et al., 2012 [221]	Respiratory symptoms and influenza vaccination immune response	Randomized, double-blind, placebo-controlled, parallel-group, longitudinal study; 6 mo; *n* = 737; Belgium	Fermented milk containing at least 6.5 x 10^9^ CFU/bottle of LcS	The daily consumption of a fermented milk drink that contains LcS has no statistically or clinically significant effect on the protection against respiratory symptoms.
Nagata et al., 2011 [122]	Reduce fever in norovirus gastroenteritis	Open-label, case-controlled, longitudinal study; 3 mo; *n* = 77; Japan	Fermented milk containing 4 × 10^10^ CFU/bottle of LcS	Continuous intake of LcS-fermented milk could positively contribute to the alleviation of fever caused by norovirus gastroenteritis by correcting the imbalance of the intestinal microflora peculiar to the elderly, although such consumption could not protect them from the disease.
An et al., 2010 [223]	Improve constipation	Single-arm, open-label, pre–post study; 2 wk; *n* = 19; South Korea	Probiotic containing *Lactobacillus acidophilus* CBT, *Pediococcus pentosaceus* CBT, and *Bifidobacterium longum* SPM 1205 (3.0 × 10^11^ CFU/g)	Lactic acid bacteria increased defecation habits such as frequency of defecation, amount, and state of stool when added to the standard treatment regimen for nursing-home residents with chronic constipation.
Lahtinen et al., 2009 [224]	Improve gastrointestinal composition	Randomized, placebo-controlled, longitudinal study; 6 mo; *n* = 66; Finland	Fermented oat drink containing 10^9^ CFU/ml of *Bifidobacterium longum* 46 (DSM 14583) and *B. longum* 2C (DSM 14579)	*Bifidobacterium* may be modulated by probiotic administration in elderly subjects.
Ouwehand et al., 2008 [225]	Improve immune system	Randomized, double-blind, placebo-controlled, longitudinal study; 6 mo; *n* = 209; Finland	Oat-based drink supplemented with 10^9^ CFU/mL of both *Bifidobacterium longum* 2C (DSM 14579) and 46 (DSM 14583)	This indicates that modulation of fecal *Bifidobacterium* may provide a means of influencing inflammatory responses.

Abbreviations: BB12, *Bifidobacterium animalis* subsp. *lactis* BB-12; CFU, colony-forming units; LcS, *Lactobacillus casei* strain Shirota; LGG, *Lactobacillus rhamnosus* GG; MDRO, multidrug-resistant organism.

Emerging strategies are now shifting toward precision probiotics—personalized interventions tailored to an individual’s baseline microbiota and metabolic profile. These approaches aim to restore specific microbial functions or occupy ecological niches relevant to host health, such as promoting lactate or butyrate production. The growing recognition of responders versus nonresponders to probiotic supplementation further highlights the need for microbiome-informed selection criteria supported by multiomic tools and predictive algorithms to enhance the efficacy of interventions in aging populations [227].

Despite these advances, current evidence is insufficient to support the routine clinical use of pro-, pre-, or postbiotics in institutionalized settings. Nevertheless, their use is increasing, often outside of formal protocols, which underscores the need for rigorous safety standards. Although adverse events are rare, potential risks include systemic infections, production of harmful metabolites, horizontal gene transfer of antibiotic resistance, unintended immune effects, or adverse reactions to formulation excipients [228,229]. Therefore, careful screening of vulnerable individuals and the implementation of standardized protocols are essential to minimize risks and ensure safe use in this population.

### Exercise and diet as synergistic strategies to support the gut microbiome and healthy aging

Along with diet, physical activity is a key pillar of healthy aging and is closely intertwined with the nutritional needs of elderly. Regular physical activity has well-documented benefits, including improved mobility, reduced risk of sarcopenia and frailty, enhanced bone health, fall prevention, and cognitive support [230]. It is also associated with broader multisystem improvements, such as increased muscle protein synthesis, enhanced respiratory capacity, reduced blood pressure, greater neurogenesis, improved bone density and muscle mass, and reduced adiposity [231].

Age-related decline in physical activity is being increasingly recognized as a factor contributing to alterations in the gut microbiome. Although current knowledge remains limited—partly because of methodological heterogeneity and lack of standardization in sample collection and analysis [232]—emerging evidence suggests that sedentary behavior negatively affects gut microbial composition, whereas regular physical activity may exert beneficial modulatory effects [233,234].

Although data specifically focused on institutionalized populations are scarce, small-scale interventions in elderly have shown promising results. For instance, Taniguchi et al. [235] reported that a 5-wk aerobic program increased the abundance of the genus *Oscillospira* and reduced *C. difficile* in older men [235]. Similarly, an 8-wk combined exercise program in older women led to increases in *Lachnospiraceae*, *Roseburia*, *Mitsuokella*, and *Akkermansia* and decreases in *Bacillota*, *Bacteroidaceae*, *Clostridioides*, and *Escherichia* [236]. Notably, exercise appears to exert more pronounced microbiome benefits in overweight elderly than in those of normal weight [237].

Several factors inherent to the nursing-home environment contribute to the low levels of physical activity observed among residents. Daily routines in these settings are often rigid, governed by strict protocols and social norms that limit opportunities for spontaneous movement or exercise. Additionally, staff may be reluctant to promote physical activity because of perceived risks, limited training, low confidence in benefits, insufficient institutional support, heavy workloads, and high staff turnover [238]. Environmental barriers such as restricted physical space can further compound the problem, especially given the high prevalence of physical and cognitive impairments among residents [239]. In this context, implementing cutomized and supervised exercise programs in long-term care facilities could offer substantial health benefits—not only by improving physical function and quality of life but also by supporting gut health through microbiota modulation.

Moreover, growing evidence supports the synergistic effects of diet and exercise on the gut microbiome, particularly in elderly. A systematic review by Mailing et al. [240] concluded that exercise-induced shifts in microbial composition, such as increases in *Akkermansia* and SCFA-producing taxa, are more robust and beneficial when accompanied by fiber-rich or prebiotic-enriched diets, with associated improvements in intestinal barrier integrity, systemic inflammation, and metabolic regulation. These interventions were associated with improved intestinal barrier integrity, reduced systemic inflammation, and better metabolic regulation. In a prospective trial, Cronin et al. [241] found that 8 wk of moderate exercise plus whey protein supplementation enhanced microbial diversity and enriched taxa linked to metabolic health. More recently, Brooks et al. [242] highlighted that combining dietary and physical activity interventions in elderly can enhance gut microbial diversity and function, potentially mitigating age-related dysbiosis and supporting host resilience.

Together, these findings reinforce the concept that integrated diet and exercise strategies offer a powerful, synergistic approach to support gut microbial resilience and promote healthier aging trajectories. These are particularly relevant in institutionalized elderly at increased risk of dysbiosis and functional decline.

Institutionalized elderly face several physiological, nutritional, and environmental challenges that affect gut microbiota composition and overall health. This review highlights how aging-related changes, such as immune dysfunction, polypharmacy, and frailty, interact with dietary limitations and care practices to shape the gut ecosystem. The evidence indicates that both macro- and micronutrient intakes, along with food processing and sensory factors, influence microbial diversity and function in long-term care settings. Moreover, features inherent to institutional life, such as standardized menus, restricted mobility, and limited personalization, further contribute to gut microbiota dysfunction and undernutrition.

A growing body of evidence supports the feasibility of microbiota-informed, multimodal strategies, including dietary adaptations, physical activity, targeted supplementation, and digital tools, to enhance health and resilience in institutionalized elderly. However, their implementation remains limited, underscoring the need for translational research and context-specific solutions.

First, there is a pressing need for well-designed longitudinal studies and controlled interventions specifically tailored to nursing-home settings. These studies should take into account the unique physiological, psychological, and environmental profiles of institutionalized elderly who often present with complex comorbidities and medication regimens. Particularly promising interventions are multimodal interventions that combine nutritional strategies with physical activity, cognitive stimulation, and psychosocial engagement. These combined approaches may offer greater benefits than single interventions and are especially relevant in long-term care where residents’ needs are complex and multidimensional.

Another priority is the development of standardized protocols for microbiome sampling, storage, sequencing, and functional analysis, particularly for frail populations or those with polypharmacy. Harmonizing these protocols would enhance comparability across studies and facilitate meta-analyses that could be used to develop guidelines and policies.

Translating microbiome science into practice will require the use of implementation science frameworks to assess the feasibility, cost-effectiveness, and scalability of interventions in real-world care environments. This includes understanding institutional constraints, staff capacities, and resident engagement.

Finally, the responsible integration of digital health and AI tools into personalized nutrition holds great promise but also presents challenges. These technologies must be developed ethically and inclusively, with attention to digital literacy, data privacy, and staff training to ensure equitable impact and avoid widening health disparities.

As highlighted in the lower section of Figure 2, addressing these interconnected challenges and knowledge gaps will require coordinated and cross-disciplinary efforts, sustained investment, and a strong commitment to translational outcomes that promote autonomy and quality of life for institutionalized elderly.

## Author contributions

The authors’ responsibilities were as follows – CJ-A, NM: methodology, writing, review, and editing; MVM-A: conceptualization, methodology, writing, supervision, review, and editing; and all authors: read and approved the final manuscript.

## Funding

The authors acknowledge funding from the Spanish Ministry of Science, Innovation, and Universities (Project PID2023-148419OB-I00, MICIN).

## Conflict of interest

The authors report no conflicts of interest.
